# Lateral Abdominal Wall Hibernoma: A Rare Case From the Maldives

**DOI:** 10.7759/cureus.111221

**Published:** 2026-06-21

**Authors:** Ahmed R Zubi, Aminath Shafeenaz Moosa, Imran M Adam, Ghada M Yousef

**Affiliations:** 1 General Surgery, Indira Gandhi Memorial Hospital, Malé, MDV; 2 Pathology, Indira Gandhi Memorial Hospital, Malé, MDV

**Keywords:** abdominal wall, benign soft tissue tumor, brown adipose tissue, hibernoma, internal oblique muscle, soft tissue mri

## Abstract

Hibernoma is a rare, benign soft tissue tumor originating from residual fetal brown adipose tissue and derives its name from its histological resemblance to brown fat in hibernating animals. These tumors most commonly occur in adults during the third and fourth decades of life, typically in the thigh, though other sites include the axilla, shoulder, back, neck, and retroperitoneum. Complete surgical excision is the treatment of choice and carries an excellent prognosis.

We report a case of symptomatic hibernoma arising in the lateral abdominal wall of a 35-year-old Maldivian woman who presented with an eight-month history of a progressively enlarging, dull aching mass in the right lumbar region. Magnetic resonance imaging (MRI) revealed a well-defined 9.7 × 6.6 × 5.7 cm soft tissue mass within the myofascial plane between the external and internal oblique muscles. The mass was completely excised, and histopathological examination confirmed hibernoma, showing a well-circumscribed, lobulated mass with a tan-yellow to reddish-brown cut surface. Microscopically, sheets of polygonal cells with multivacuolated granular cytoplasm and small central nuclei were observed, with no atypia, necrosis, or mitotic figures. The patient remained asymptomatic at one-month follow-up.

To the best of our knowledge, this is the first reported case of hibernoma from the Maldives and one of the few cases arising from the abdominal wall musculature, with the unique feature of originating from the internal oblique muscle. This case highlights the importance of considering hibernoma in the differential diagnosis of deep-seated soft tissue tumors, particularly when imaging reveals a well-defined, heterogeneous mass with benign characteristics.

## Introduction

Hibernoma was first described by Merkel in 1906 as a “pseudo-lipoma.” In 1914, Gery introduced the term “hibernoma” based on its histological resemblance to the brown adipose tissue of hibernating animals [1,2]. This rare, benign soft tissue tumor accounts for approximately 1% of all adipose tissue tumors. It arises from residual fetal brown adipose tissue that may persist into adult life, and malignant transformation has not been reported. Hibernomas most commonly present in adults during the third and fourth decades of life. The thigh is the most frequent location, although these tumors may also arise in the subcutaneous or intramuscular layers of the axilla, shoulder, back, neck, chest, and arm, as well as in intraosseous and retroperitoneal sites [1-3].

Clinically, hibernomas are often incidental findings and manifest as slow-growing, firm lesions within the subcutaneous or intramuscular tissue. They may cause mild pain, discomfort, or pressure symptoms. The differential diagnosis includes fat necrosis, angiolipoma, giant cell tumor, rhabdomyosarcoma, lipoma, and liposarcoma [1,4].

Magnetic resonance imaging (MRI) is the imaging modality of choice [5]. Gross examination reveals a well-circumscribed yellow to brown mass, while microscopic examination demonstrates sheets of polygonal cells with granular, eosinophilic cytoplasm containing multiple small vacuoles and a centrally located nucleus [6]. Complete surgical excision is the treatment of choice and is associated with an excellent prognosis [1,7].

We report a rare case of symptomatic hibernoma arising in the lateral abdominal wall of a 35-year-old woman. The clinical, imaging, and histological findings are described, and the patient was successfully managed with surgical excision. A comprehensive literature review is also provided. To the best of our knowledge, this is the first reported case of hibernoma from the Maldives. This report underscores the importance of considering hibernoma in the differential diagnosis of deep-seated soft tissue tumors.

## Case presentation

A 35-year-old Maldivian woman with no significant past medical history presented with an eight-month history of a progressively enlarging, dull aching mass in the right lumbar region. The pain was initially intermittent but became constant over time. There were no overlying skin changes, constitutional symptoms, weight loss, or alterations in bowel or urinary habits. Her surgical history was notable for bilateral axillary fat pad excision performed 10 years prior to presentation.

One month before presenting to our institution, she underwent ultrasonography (USG) at a regional hospital, which suggested a deep subcutaneous lipoma. The report was reviewed by the treating clinician. Subsequent MRI revealed a well-defined soft tissue mass measuring 9.7 × 6.6 × 5.7 cm within the myofascial plane of the right lateral abdominal wall. The lesion was located between the external and internal oblique muscles and appeared inseparable from the internal oblique muscle. On MRI, the mass was hypointense on T1-weighted imaging and hyperintense on T2-weighted imaging relative to adjacent muscles, with mild heterogeneity. These features were considered likely benign (Figure 1).

**Figure 1:**
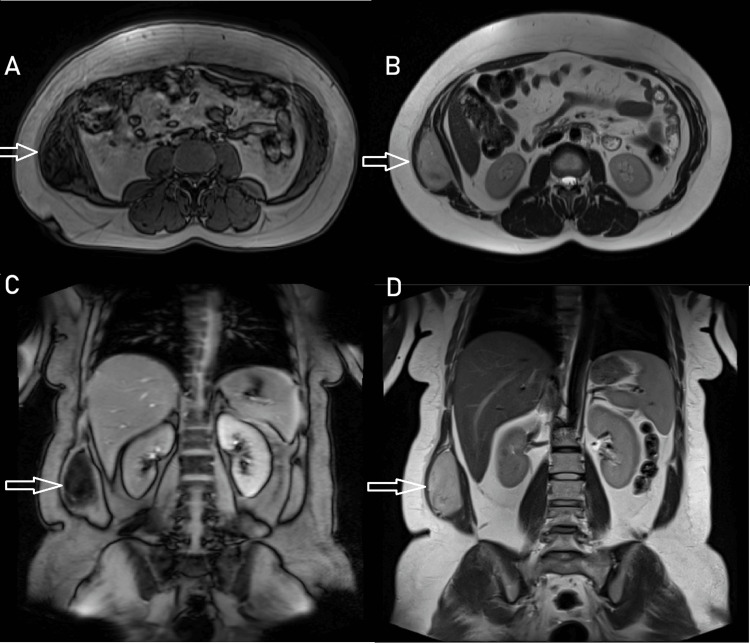
Abdominal MRI. MRI of the right lateral abdominal wall demonstrating a large, well-defined, nearly circumscribed soft tissue lesion within the myofascial plane (white arrows). (A) T1-weighted axial view. (B) T2-weighted axial view. (C) T1-weighted coronal view. (D) T2-weighted coronal view. MRI: magnetic resonance imaging

On physical examination in our outpatient department, the patient appeared well, with a body mass index (BMI) of 32 kg/m². Local examination revealed a deep-seated, firm, slightly tender lump in the right lumbar region, measuring approximately 10 × 7 cm. The mass was not adherent to the skin, which moved freely over it. However, the mass demonstrated restricted mobility and became less prominent during the contraction of the underlying abdominal wall musculature. No cough impulse was elicited. Routine hematological and biochemical investigations were within normal limits.

Under general anesthesia, the mass was excised via a transverse incision of approximately 10 cm in the right lumbar region. After splitting the external oblique muscle, the lesion was identified within the internal oblique muscle, extending downward and forward from the tip of the 12th rib (Figure 2). The lesion was dissected from the surrounding tissue using monopolar diathermy. A clear dissection plane was observed between the mass and the surrounding muscles, except at the upper pole, where a fibrous band extended toward the tip of the 12th rib. This band was divided with monopolar diathermy. The vascular pedicle was ligated with a Vicryl 2-0 suture. Subsequently, the mass was completely excised. The muscle was then approximated, and the wound was closed in layers. The patient was discharged the following day and remained asymptomatic at a five-day in-person follow-up and at a one-month telephone follow-up.

**Figure 2:**
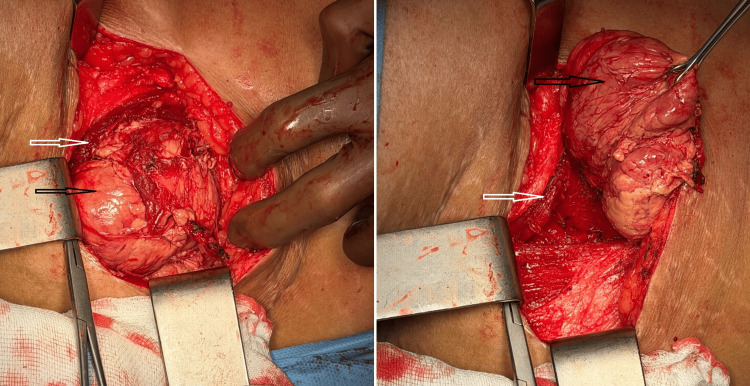
Intraoperative photographs. Intraoperative photographs of the right lumbar region. The hibernoma (black arrow) is seen deep to the external oblique muscle (white arrow). The patient’s head is oriented to the right of each image.

Histopathological examination revealed a well-circumscribed, encapsulated lesion measuring 8.0 × 7.5 × 2.5 cm. The cut surface was lobulated, tan-yellow to reddish-brown, greasy, soft, and spongy, with prominent feeding vessels (Figure 3). Microscopically, the tumor was composed of large polygonal cells arranged in sheets. These cells exhibited multivacuolated, granular cytoplasm with small, centrally located nuclei, admixed with variable numbers of univacuolated cells. No significant cytological atypia, necrosis, or mitotic figures were identified (Figure 4). To definitively exclude the primary histological mimics of hibernoma, a targeted immunohistochemical panel was performed. Diffuse positive staining for S100 protein confirmed adipocytic lineage, while the complete absence of SOX10 effectively ruled out a granular cell tumor, which typically co-expresses both markers. Adult rhabdomyoma was excluded by negative desmin staining. Furthermore, absolute negativity for MDM2 and CDK4 reliably excluded well-differentiated liposarcoma. A minimal Ki-67 proliferation index (up to 5%) and a benign vascular network highlighted by CD34 and smooth muscle actin (SMA) further supported the diagnosis. Collectively, this immunoprofile conclusively distinguishes hibernoma from all granular and malignant mimics. Immunohistochemical staining was performed at an external laboratory; therefore, the images were not available for inclusion.

**Figure 3:**
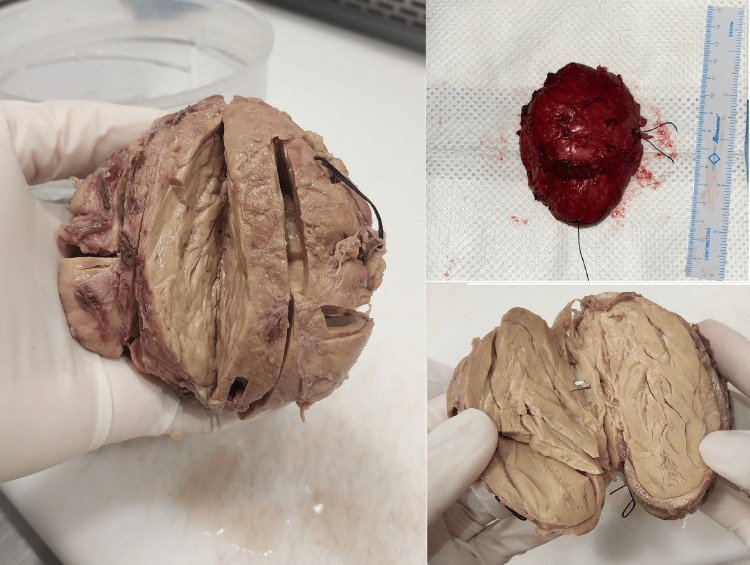
Gross specimen. Gross specimen demonstrating a well-circumscribed, encapsulated, lobulated, soft tissue mass with a tan-yellow to reddish-brown cut surface.

**Figure 4:**
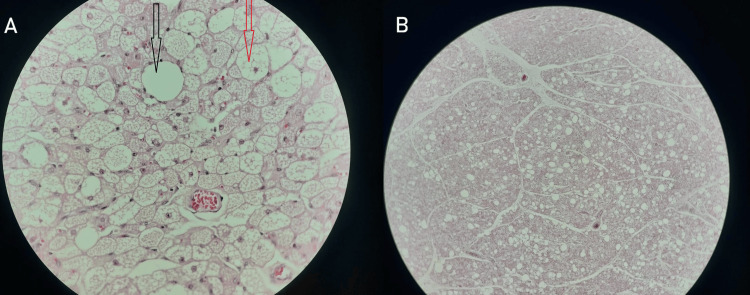
Microscopic photographs. Hematoxylin and eosin (H&E)-stained sections showing sheets of polygonal brown fat cells with multivacuolated granular eosinophilic cytoplasm and small central nuclei (red arrow), admixed with variable numbers of univacuolated cells (black arrow), and the absence of atypia, necrosis, or mitotic activity. A, ×40; B, ×10.

## Discussion

Although brown fat starts to involute after birth and is replaced by white fat, neoplastic transformation is more common in adulthood with a mean age of approximately 38-59 years [2,3]. However, infantile hibernoma has also been reported, as has hibernoma in patients as old as 85 years [2,8]. The tumor commonly involves the thigh, shoulder, back, neck, chest, arm, abdominal cavity/retroperitoneum, and intraosseous sites [2,3]. Hibernoma arising from the anterior abdominal wall muscle is extremely rare; to the best of our knowledge, only one case has been reported. In that case, the hibernoma was located in the external oblique muscle, whereas in the present case, it arose from the internal oblique muscle [4]. Both patients were in their 30s. In the largest study to date, Furlong et al. reported four cases of anterior abdominal hibernoma, but they did not specify whether these were intramuscular or subcutaneous [3].

Hibernoma is a benign tumor that presents as a lump when arising in subcutaneous or muscular tissue and may cause discomfort or pain primarily due to pressure on adjacent structures. However, deep-seated hibernomas in the retroperitoneal or intraosseous position are usually discovered incidentally [2,9]. Complications are rare. The physical examination reveals a soft to firm mass, which is mobile in the subcutaneous tissue but has restricted mobility for intramuscular lesions. It is usually not tender and does not cause skin changes. Its size ranges from 1 to 24 cm, but it is often 7-13 cm at diagnosis [3]. This case presentation corresponded with the literature for the slowly growing, mildly painful mass, but it has an exceptionally rare location in the oblique abdominal muscles. The discrepancy between MRI (9.7 × 6.6 × 5.7 cm) and post-excision measurements (8.0 × 7.5 × 2.5 cm) is likely attributable to post-excisional tumor shrinkage due to the loss of vascular volume, differences in specimen orientation, and potential motion artifact on MRI leading to the overestimation of the lesion. The patient had a history of axillary fat pad excision performed 10 years before presentation. This finding was considered incidental, as no clear association between axillary fat pad and hibernoma has been reported in the literature.

Imaging is the initial evaluation modality for hibernoma. Most cases are initially assessed with USG, followed by computed tomography (CT) and/or MRI. A positron emission tomography (PET) scan is rarely performed for intramuscular or subcutaneous hibernoma [7]; however, in intraosseous hibernoma, ¹⁸F-FDG PET, bone scintigraphy, and single-photon emission computed tomography (SPECT) are frequently utilized [2].

On imaging, the tumor typically presents as a regular, well-defined mass: hyperechoic on USG with hypervascularity on Doppler imaging, hypodense relative to muscle on CT, and hypointense to isointense on T1-weighted MRI, with hyperintensity on T2-weighted MRI and incomplete fat saturation. The lesion may appear heterogeneous with irregular enhancement and may demonstrate a prominent central vessel. MRI is considered the more accurate modality for delineating the exact size, tissue plane, and nature of the soft tissue mass [5,10]. On FDG PET, hibernoma exhibits mild to moderate increased tracer uptake, attributable to hypervascularity and elevated metabolic activity [5]. Although plain radiography is nonspecific, it may indirectly demonstrate a soft tissue shadow or the displacement of adjacent structures, prompting further imaging [8,10]. In the present case, USG was initially interpreted as a deep‑seated lipoma in the right flank, prompting subsequent MRI. In accordance with the literature, MRI revealed a well‑defined soft tissue mass with slight heterogeneity, appearing hypointense on T1‑weighted imaging and hyperintense on T2‑weighted imaging, features consistent with a benign lesion. This imaging profile helped differentiate the hibernoma from a lipoma, which typically appears hyperintense on T1‑weighted imaging and loses signal on fat suppression sequences.

Although MRI features of hibernoma are not specific and vary according to the lesion’s fat component, they can nonetheless indicate the benign nature of the mass [5]. In the reported case, the surgical team relied on the absence of malignant features on MRI and proceeded to complete excision rather than core needle biopsy. Although biopsy plays an important role in planning the extent of surgery, the literature demonstrates mixed approaches, with core biopsy omitted in many cases [8,9,11,12]. Furthermore, the vascular nature of the lesion may predispose to bleeding complications following core biopsy. That notwithstanding, Papathanassiou et al. reported 12 cases that underwent core biopsy without complications [5].

Owing to the rarity of hibernomas, a significant number of cases are either not diagnosed at the time of surgery or are misdiagnosed as lipomas [13]. Lipomas differ from hibernomas by being more homogeneous with fat-characteristic features and lacking contrast enhancement [5]. Other important differential diagnoses include angiolipoma, hemangioma, and liposarcoma. Kojima et al. found that liposarcoma may harbor foci of hibernoma-like histology [14].

Excision is the preferred treatment for hibernoma. Although recurrence is very rare, complete excision is important, as some papers report recurrence after the incomplete removal of the lesion [3,15]. Given that hibernoma is a benign lesion with no metastatic potential, conservative management has been offered to asymptomatic patients [1,6]; however, long-term follow-up data in these reports are lacking. In cases of intraosseous hibernoma, conservative management is the predominant approach, as surgical excision may entail considerable morbidity. Less invasive methods, such as percutaneous thermal ablation, have also been reported. Conversely, extensive resection, such as nephrectomy, has been performed in cases where imaging suggested a malignant lesion [9]. In the present case, simple excision was performed, consistent with the majority of the literature, as imaging indicated a benign lesion. In a previously reported case of external oblique muscle hibernoma, the lesion was removed with a cuff of muscle, followed by simple closure [4]. Hibernomas are typically well-encapsulated and easily separated from surrounding structures; therefore, the excision of a cuff of normal tissue is not routine practice [11,16].

This case report has several limitations. First, the unavailability of immunohistochemistry images reduces the educational value of the report. Second, the follow-up period was limited to one month, which precludes meaningful comment on recurrence or long-term complications.

## Conclusions

Hibernoma, though rare, merits consideration in the differential diagnosis of deep-seated or slowly enlarging subcutaneous and intramuscular soft tissue masses. While most commonly arising in the limbs, hibernoma can develop in any fat-containing tissue. We present the first documented case from the Maldives, with the unique feature of arising from the internal oblique muscle. The clinical assessment of mass mobility offers valuable insight into its anatomical plane: intramuscular lesions exhibit greater mobility perpendicular to the muscle’s long axis than parallel to it, with the complete restriction of movement during active muscle contraction. Preoperative imaging, particularly MRI, aids in lesion characterization and supports a benign impression; however, definitive diagnosis remains histopathological, based on the identification of multivacuolated polygonal cells with granular eosinophilic cytoplasm. Complete surgical excision is curative and associated with an excellent prognosis. Although our one-month follow-up precludes the meaningful assessment of recurrence risk, consistent with the generally indolent behavior of hibernomas, this case reinforces the importance of clinical and pathological awareness to avoid misdiagnosis and ensure optimal patient management, particularly when tumors present at unusual anatomical sites or in underreported geographic populations.

## References

[1] Nasner D, Rincón EA, Escobar HF, Sua LF, Mera-Collazos J (2023). Lipoma-like hibernoma of the breast: a case report and literature review. Radiol Case Rep.

[2] Albashri J, Albashri A, Alhamrani M (2025). Clinical, radiological, and pathological features of intraosseous hibernoma: a systematic review of case reports and case series. Curr Oncol.

[3] Furlong MA, Fanburg-Smith JC, Miettinen M (2001). The morphologic spectrum of hibernoma: a clinicopathologic study of 170 cases. Am J Surg Pathol.

[4] Baghaie H, Chan E, Francisco S, Rasheed H, Puhalla H (2021). Intramuscular abdominal hibernoma: case report of a rare tumour and a review of the literature. J Surg Case Rep.

[5] Papathanassiou ZG, Alberghini M, Taieb S, Errani C, Picci P, Vanel D (2011). Imaging of hibernomas: a retrospective study on twelve cases. Clin Sarcoma Res.

[6] Gabra A, Sacasa A, Baig M, Kleinstein E (2023). Lipoma-like hibernoma: a rare soft tissue tumor-a case series and review of the literature. J Surg Case Rep.

[7] Whalen M, Quach T, Greenberg D, Reisenbichler E, Shoela R (2025). Incidental hibernoma with an atypical presentation: diagnostic approach, pathologic correlation, and lessons learned. Cureus.

[8] Letica-Kriegel AS, Cramm SL, Moran CJ, Dunn-Albanese L, Jobbagy S, Westra SJ, Ryan D (2022). Mesenteric hibernoma in a pediatric patient present on prenatal ultrasound. J Pediatr Surg Case Rep.

[9] Streich L, Yang X (2020). Hibernoma with inflammatory features: a description of a new variant. Hum Pathol Case Rep.

[10] Park JY, Yi SY, Lee JY, Kwon TJ (2022). A case report of axillary hibernoma: US, CT, MR, and histopathologic findings. Taehan Yongsang Uihakhoe Chi.

[11] Gauci K, Debono F, Gauci F, Panzavecchia F (2023). A case of a rare hibernoma tumour and a brief literature review. Int J Res Orthop.

[12] Moosakutty S, Edakkattu AF (2026). Intermuscular hibernoma of the thigh: a diagnostic and surgical challenge managed with preoperative embolization. Cureus.

[13] Yu-Wei C, Wen-Chieh L (2025). Hibernoma: experience of a tertiary center in Taiwan. J Taiwan Soc Plast Surg.

[14] Kojima N, Komiyama M, Shinoda Y, Watanabe S, Yatabe Y, Kawai A, Yoshida A (2022). Liposarcoma with hibernoma-like histology: a clinicopathologic study of 16 cases. Am J Surg Pathol.

[15] Lele SM, Chundru S, Chaljub G, Adegboyega P, Haque AK (2002). Hibernoma: a report of 2 unusual cases with a review of the literature. Arch Pathol Lab Med.

[16] Elafram R, Khessairi N, Romdhane MB, Sghaier M, Hamdi A (2023). The lipoma-like hibernoma: a case report of a rare entity. Radiol Case Rep.

